# Cecropins from *Plutella xylostella* and Their Interaction with *Metarhizium anisopliae*

**DOI:** 10.1371/journal.pone.0142451

**Published:** 2015-11-06

**Authors:** Lina Ouyang, Xiaoxia Xu, Shoaib Freed, Yanfu Gao, Jing Yu, Shuang Wang, Wenyan Ju, Yuqing Zhang, Fengliang Jin

**Affiliations:** 1 College of Agriculture, South China Agricultural University, Guangzhou, 510642, P. R. China; 2 Department of Entomology, Faculty of Agricultural Sciences and Technology, Bahauddin Zakariya University, Multan, 60800, Pakistan; Institute of Plant Physiology and Ecology, CHINA

## Abstract

Cecropins are the most potent induced peptides to resist invading microorganisms. In the present study, two full length cDNA encoding cecropin2 (Px-cec2) and cecropin3 (Px-cec3) were obtained from *P*. *xylostella* by integrated analysis of genome and transcriptome data. qRT-PCR analysis revealed the high levels of transcripts of Px-cecs (Px-cec1, Px-cec2 and Px-cec3) in epidermis, fat body and hemocytes after 24, 30 and 36 h induction of *Metarhizium anisopliae*, respectively. Silencing of Spätzle and Dorsal separately caused the low expression of cecropins in the fat body, epidermis and hemocytes, and made the *P*.*xylostella* larvae more susceptible to *M*. *anisopliae*. Antimicrobial assays demonstrated that the purified recombinant cecropins, i.e., Px-cec1, Px-cec2 and Px-cec3, exerted a broad spectrum of antimicrobial activity against fungi, as well as Gram-positive and Gram-negative bacteria. Especially, Px-cecs showed higher activity against *M*. *anisopliae* than another selected fungi isolates. Scanning electron microscopy (SEM) and transmission electron microscopy (TEM) revealed that cecropins exerted the vital morphological alterations to the spores of *M*. *anisopliae*. Based on our results, cecropins played an imperative role in resisting infection of *M*. *anisopliae*, which will provide the foundation of biological control of insect pests by using cecorpins as a target in the future.

## Introduction

All organisms living in the natural world might have the risk of being pathogen invasion; insects are not of the exception. Insects living in complex environment combat infections, which relies mostly on the innate immunity comprising of cellular and humoral immune responses [1]. The immune deficiency (IMD) triggered by Gram-negative bacteria, including the upper pattern recognition receptors i.e., PGRP-LC and PGRP-LE, and the downstream signaling molecules i.e., dFADD, Dredd, dTAK1, dIKK complex and Relish [2]. Toll pathways are activated by Gram-positive bacteria and fungi [3,4] in which certain gene expression products are released into the hemolymph, which resist the invasion of pathogens.

The typical effectors in the intracellular signal transduction pathways are antimicrobial peptides (AMPs) [5]. Most of these are small cationic molecules consisting of 12–50 amino acids [6,7], which are able to disrupt bacterial membranes via non-specific electrostatic interactions with the membrane lipids. AMPs have broad-spectrum antibacterial property [8], active against bacteria and/or fungi, as well as some parasites and viruses [9]. According to the molecular structure, insect AMPs can be classified into four families: cysteine-loss residues peptides (cecropin and moricin), cysteine-rich peptides (defensin and drosomycin), proline-rich peptides (apidaecin, drosocin, and lebocin), and glycine-rich peptides/proteins (attacin and gloverin) [10–12]

Cecropins are one of basic antimicrobial peptides produced by insects [13]. Cecropin has been initially isolated from the hemolymph of *Hyalophora cecropia* during 1980 [14], subsequently, different forms of cecropins have been isolated from other insect species, such as *Bombyx mori* [15], *Ceratitis capitata* [16], *Glossina morsitans morsitans* [17], *Manduca sexta* [18], *Trichoplusia ni* [19] and also from one mammal, the pig [20]. In *Drosophila melanogaster*, the cecropin multigenes family consists of both four functional genes (Cecropin A1, A2, B and C), two pseudo-genes (Cecropin 1 and 2) and the functional genes coding for cecropins [21,22]. On bacterial infection, all functional genes are expressed mainly in the fat bodies at different developmental stages [23]. Cecropin A1 and A2 are mainly expressed in larvae and adults, while, B and C are mostly expressed during the pupal stage [22]. At low concentrations (0.1 to 5 μM), cecropins exhibit lytic antibacterial activities against several Gram-negative and Gram-positive bacteria, but not against eukaryotic cells [24–26].

The diamondback moth (DBM), *P*. *xylostella* (Lepidoptera: Plutellidae) is a worldwide pest, causing serious damage about 4–5 billion USD annually to cruciferous crops [27], such as cabbage, broccoli, and cauliflower [28]. Due to its high fecundity, overlapping generations, genetic plasticity and selection pressure to various insecticides [29–31], entomopathogenic fungi such as *M*. *anisopliae* has been used as a biological control agent for a long time to reduce pesticide residues and ensure food safety [32]. Cecropins are the terminal effectors which can be activated by the infection of entomopathogenic fungus. However, the mechanism of the interaction of *M*. *anisopliae* with the innate immunity of *P*. *xylostella* is unclear.

By decoding the genomic sequence of *P*. *xylostella* [33], more immune-related genes will be found and further research will be needed to confirm the modulation of cecropins expression in *P*. *xylostella*. The objective of the current study was to investigate, whether cecropins have any role against the successful invasion of *M*. *anisopliae* in *P*. *xylostella*, and if so what is their impact and mechanism in *P*. *xylostella*?

## Materials and Methods

### Insects and microorganisms

*P*. *xylostella* were reared on an artificial diet at 25 ± 2°C in 14 h: 10 h light: dark photoperiod and 60–70% relative humidity. *D*. *melanogaster* Schneider S2 cells were kindly provided by Prof. Wenqing Zhang (Sun-yat Sen University, Guangzhou, China) and were maintained in an incubator at 27°C with Schneider's *Drosophila* medium (Gibco, USA) supplemented with 10% fetal bovine serum (FBS) (Gibco, USA).

*Salmonella choleraesuis* (Smith), *Escherichia coli* DH5α, *Pseudomonas fluorescens* (Flugge), *Bacillus cereus* (Flugge), *Staphylococcus aureus* Rosenbach and fungi, *Botrytis cinerea* (Klotzsch), *Penicillium crustosum* Thom, *Peronophythora litchi*, *Colletotrichum orbicular* Berk, *Fusarium oxysporum* Schlecht, *Colletotrichum gloeosporioiees* Penz. were obtained from the Research Institute of Microbiology, Guangzhou, China, while the insect pathogenic fungus, *M*. *anisopliae* (MaQ 10) was provided by Dr. Qiongbo Hu (South China Agricultural University, Guangzhou, China), which was kept in China Center for Type Culture Collection (No. CCTCCM 208173).

The bacteria *S*. *aureus*, *B*. *cereus*, *E*.*coli* DH5α, *P*. *fluorescent*, *S*. *choleraesuis* were grown on LB broth at 37°C to mid-log bacteria(2–7×10^5^CFU/ml). The fungi, *M*. *anisopliae*, *B*. *cinerea*, *P*. *crustosum*, *P*. *litchi*, *C*. *orbicular*, *F*. *oxysporum*, and *C*. *gloeosporioiees* were grown on potato dextrose agar (PDA) plates and incubated at 26 ± 2°C for 10 days. The conidia were then harvested in deionized water containing 0.05% Tween-80 to a final concentration of 1×10^9^ conidia/ml. Spore viability was determined before preparation of final concentration by spreading 0.2 ml suspension on PDA and estimating the number of germinated propagules after 24 h of incubation at room temperature.

### Cloning of cecropin genes

Total RNA was extracted from the fat body of each instar *P*. *xylostella* after 24h treatment with *M*. *anisopliae* using Trizol reagent according to the manufacturer’s protocol (Invitrogen, USA). First-strand cDNA was synthesized with 2μg of total RNA in combination with oligo-dT_18_ primer and Super-script III reverse transcriptase (TaKaRa, Japan) was used to remove the genomic DNA. Px-cec2 and Px-cec3 Unigenes sequences were obtained from *P*. *xylostella* transcriptome and the PCR reactions were performed according to the following conditions: 5 min at 94°C, 30 cycles at 94°C for 30 sec, 55°C (Px-cec2) or 56°C (Px-cec3) for 30 sec, 72°C for 30 sec and 8 min at 72°C. 2 μg of mRNA was used to prepare the 5′- and 3′-RACE cDNAs using the SMART RACE cDNA Amplification Kit (TaKaRa, Japan). 3′—UTR region was amplified by 3′-RACE using the following touchdown PCR: 5 cycles of 94°C 30 sec, 72°C 3 min; 5 cycles of 94°C 30 sec, 70°C 30 sec, 72°C 1 min; 25 cycles of 94°C for 30 sec, 62°C (Px-cec2) or 65°C (Px-cec3) for 30 sec, and 72°C for 1 min, while for 5′—UTR, annealing temperature was changed to 65°C (Px-cec2) or 63°C (Px-cec3). All primers Px-cec2-F1/Px-cec-R and Px-cec3-F1/ Px-cec-R for 3′ -UTR and Px-cec-F/Px-cec-R2, Px-cec-F/Px-cec-R2 for 5′ -UTR are shown in Table 1.

**Table 1:** List of Primers and their sequences used in experiment.

Gene name	Sequence(5’-------3’)	function
Px-cec-R	CTAATACGACTCACTATAGGGCAAGCAGTGGTATCAACGCAGAGT	UPM
Px-cec-F	CTAATACGAC TCACTATAGGGC	UPM
Px-cec2-F1	CAGGTGGAATCCGTTCAA	Px-cec2 3 ′ RACE PCR
Px-cec2-R2	TGGAGTTGGCTTGTCCTATC	Px-cec2 5 ′RACE PCR
Px-cec3-F1	GCTCCCAGGTGGAAAGGC	Px-cec3 3 ′ RACE PCR
Px-cec3-R2	CTCCATCCCGGATGTGTCGTCC	Px-cec3 5 ′RACE PCR
Actin-qF	TGGCACCACACCTTCTAC	qRT-PCR
Actin-qR	CATGATCTGGGTCATCTTCT	qRT-PCR
Px-cec1-qF	GCCAAGGTGGAAGCCGTTTA	qRT-PCR
Px-cec1- qR	TATAGAAGTGGCTTGTCCGATGA	qRT-PCR
Px-cec2-qF	TTCGTGTTGGTGGCGGTATTC	qRT-PCR
Px-cec2-qR	GCAGGTCTAGCGATGGAGTTG	qRT-PCR
Px-cec3-qF	TACTTCTTCTTCACGGTTGTCG	qRT-PCR
Px-cec3-qR	CCCAATATGCTGGATGCTTGTC	qRT-PCR
GFP-F	GGATCCTAATACGACTCACTATAGGAAGGGCGAGGAGCTGTTCACCG	dsGFP
GFP-R	GGATCCTAATACGACTCACTATAGGCAGCAGGACCATGTGATCGCGC	dsGFP
Spa-RNAiF	GGATCCTAATACGACTCACTATAGGACGCTGCCTCCAACGCTTCT	RNAi
Spa-RNAiR	GGATCCTAATACGACTCACTATAGGTCCTCGCATACCAGTCCCT	RNAi
Dor-RNAiF	GGATCCTAATACGACTCACTATAGGCTGAAGCGAAAGCGACAGAAACC	RNAi
Dor-RNAiR	GGATCCTAATACGACTCACTATAGGCTGCTGCGCCATAGGAGCCATA	RNAi
Px-cec1-ExpF	GCG*AGATCT*AAGCCGTTTAAAAAATTGGA	Recombinant expression
Px-cec1-ExpR	GC*CTCGAG*TTTGCCAGTAGGTCTGGCTATAG	Recombinant expression
Px-cec2-ExpF	GCG*AGATCT*AAATTGGAGCGAGTGGGACAGC	Recombinant expression
Px-cec2-ExpR	GC*CTCGAG*CGGCAGGTCTAGCGATGGAGTT	Recombinant expression
Px-cec3-ExpF	GCG*AGATCT*TACTTCTTCTTCACGGTTGTCG	Recombinant expression
Px-cec3-ExpR	GC*CTCGAG*TCCCATTATGCTGGATGCTTGT	Recombinant expression

Spa, Spätzle; Dor: Dorsal; *AGATCT*, restriction enzyme *Bgl* II; *CTCGAG*, restriction enzyme *Xho* I

### Sequence analysis

The cDNA sequences of cecropins were analyzed with bioinformatics analysis tools. Homology searches of cDNA were performed by using BLAST (http://blast.ncbi.nlm.nih.gov/Blast.cgi). The translation of Px-cec2, Px-cec3 and the deduced amino acid sequence was performed with ExPASY (http://www.expasy.ch/) while, the sequence alignment was performed by Clustal X. 2.0 (http://www.ebi.ac.uk/tools/clustalw2). The signal peptide was analyzed by SignalP (http://www.expasy.ch/SingalP) and the theoretical isoelectric point (pI) was calculated by the compute pI software (http://www.expasy.org/tools/pi-tool.html). A neighbor-joining phylogenetic tree was constructed based on the amino acids sequence by using MEGA 6.0.

### qRT-PCR of cecropins

To assess the mRNA expression level of Pxcecs in the 4^th^ instar *P*. *xylostella* larvae after the induction of *M*. *anisopliae* fat body, epidermis and hemocytes were isolated from *M*. *anisopliae* induced *P*. *xylostella* larvae after 6, 12, 18, 24, 30, 36, 42, 48 h and washed 3 times in 1×PBS buffer. Tween-80 was used to inject control. The cDNA was synthesized on the basis of the manufacturer’s protocol using the PrimeScript™ RT Master Mix (Perfect Real Time) (TaKaRa, Japan). The primers for qRT-PCR amplification i.e., Px-cec1-qF/qR, Px-cec2-qF/qR and Px-cec3-qF/qR (Table 1) were designed according to ORF of cecropin genes. The final reaction mixture contained 1μl of each primer, 12.5 μl of SYBR^®^ Green PCR Master Mix and 2 μl cDNA. All quantitative reactions were subjected to: 95°C for 3 min followed by 39 cycles at 95°C for 10 sec, 58°C for 30 sec, and ending with 95°C for 10 sec. While as an internal control, the partial fragment of muscle-actin gene (GenBank accession No. AB282645) was also amplified from the same cDNAs with the Actin qF/qR primers (Table 1). Melting curve analysis was applied to all reactions to ensure homogeneity of each reaction product. Three biological replications (n = 3) were performed for each reaction and the 2^-ÄÄCt^ method was used to measure the relative transcription levels.

### Recombinant expression and purification of Pxcecs

The cDNA sequence encoding mature Pxcecs was amplified by using specific primers i.e., Px-cecs-ExpF/ExpR (Table 1). The PCR fragments, purified by agarose gel electrophoresis were digested with *Bgl* II and *Xho* I enzymes, ligated into *Bgl* II /*Xho* I-digested expression vector pMT/BiP/V5-His A (Invitrogen, USA) and later on transformed into the competent DH5α cells. Whereas, the recombinant expression vectors (pMT-Cec1, pMT-Cec2, pMT-Cec3) were confirmed by DNA sequencing.

*D*. *melanogaster* Schneider S2 cells was maintained in an incubator at 27°C in Schneider cell Culture Media (Invitrogen, USA), supplemented with 10% heat-inactivated fetal bovine serum (Invitrogen, USA) containing 1% penicillin-streptomycin solution (Sigma, USA). For transfection assay, S2 cells were placed on cover slips in six-well plates overnight at a density of 1×10 ^6^ cells/mL in serum-free medium (Hyclone, USA) and were then incubated overnight, 1 μg plasmid and 6 μl Lipofectamine LTX (Gibco, USA) transfection reagent were used for transient transfection based on the manufacturer’s instructions. Construction stable S2 cell lines was performed essentially in the same manner as described previously [34].

In order to obtain recombinant cecropins, the cell culture medium containing stable S2 cells expressing Pxcecs were collected every 24 h after induction of 250 μM copper sulfate in 150-cm^2^ flasks to induce the protein. Cell culture medium was combined after 10 days collection, cell debris was removed by centrifugation at 1,000g for 10 min and cell-free medium was incubated overnight at 4°C and later on the protein was detected by 30% SDS–PAGE. Purification of recombinant cecropins was performed according to the method reported previously [35].

### RNAi of Spätzle and Dorsal

In order to minimize any off-target effect during RNAi, the gene fragments used for dsRNA synthesis comprised the sequence where a 19 bp consecutive identical cDNA sequences with Spätzle and Dorsal were not found. Double-stranded RNA (dsRNA) for Spätzle and Dorsal RNAi was prepared by using T7 RiboMAX™ Express RNAi System (Promega, USA) according to the manufacturer’s protocol. A dsRNA segment corresponding to the green fluorescent protein (GFP) was also synthesized as a negative control as described previously. dsRNAs were confirmed by gel electrophoresis in 1.0% agarose and the concentrations of dsRNAs were determined by spectrophotometer (Nanodrop 1000, Thermo Scientific) and stored at -20°C for further use.

The 4^th^ instar *P*. *xylostella* larvae were injected with 1μl dsRNA Spätzle (100ng/1μl, thirty larvae), dsRNA Dorsal (100ng/1μl, thirty larvae) and dsRNA GFP (100ng/1μl, thirty larvae) respectively. After 24 h of RNAi treatment, the repeated injection of 1μl *M*. *anisopliae* spore suspension (1×10^9^ conidia/ml) was again injected to stimulate the immune response. After 36 h of induction, qRT-PCR was performed to analyze the expression of cecropins in three tissues of *P*. *xylostella*.

### Bioassay on pathogenicity of *M*. *anisopliae*

Fourth instar larvae of *P*. *xylostella* were treated with dsRNA specific to PxSpa and PxDor as described above. After 24 h since the second dsRNA injection, the larvae were dipped into *M*. *anisopliae* spores (1×10^9^ conidia/ml) suspension for 30 sec, and then reared normally under similar conditions as described earlier. The control larvae were treated with 0.05% tween-80 solution only. Each treatment was replicated three times.

### Antimicrobial activity assays

The antimicrobial activity of the purified recombinant Pxcecs was tested against several Gram-positive and Gram-negative bacteria and fungi. The minimal growth inhibition concentration (MIC) was determined using a liquid growth inhibition assay and expressed as the lowest final concentration of the peptide at which no growth was observed [35], while the cell density was measured by monitoring the absorbance at 494 nm.

### Scanning electron microscopy

In order to clarify recombinant cecropins interacting with *M*. *anisopliae* spores. 30 μM recombinant proteins i.e., Px-cec1, Px-cec2 and Px-cec3 were incubated with mid-log phase *M*. *anisopliae* spores (1×10^9^ conidia/ml) at 37°C for 3 h and collected by centrifugation at 3,000g for 5 min. The supernatants were later on inoculated by immersion for 5 sec in 2 ml conidial suspension. The precipitates were fixed in 2.5% glutaraldehyde in 0.1 M phosphate buffer for 3 h, and washed three times with 0.1 M phosphate buffer and then dehydrated in ascending series of ethanol (50, 70, 80, 90 and 100%, 15 min each). The samples were dried at room temperature for few seconds and mounted on SEM stubs with double-sided carbon tape. Dried samples were sputtered with gold and observed with the SEM under Quanta 200 FEG at high-vacuum mode.

### Transmission electron microscopy of *M*. *anisopliae*

The *M*. *anisopliae* spores (1×10^6^ conidia/ml) were treated with 30 μM Px-cec1, Px-cec2 and Px-cec3 at 37°C for 30 min. The supernatant was collected by centrifugation (5,000g for 5 min) and cold glutaraldehyde (0.5%, v/v, in 0.1 M sodium cacodylate buffer, pH 7.4) was added for 2 h at 4°C as described previously [5]. The fixed *M*. *anisopliae* spores were observed by using a Jeol JEM 1200 transmission electron microscope operating at 80 kV.

### Statistical Analysis

All the data were presented as relative mRNA expression. The relative expression level of Cecropins was calculated by using the CFX96 Real-Time system (Bio-Rad, USA). Statistical analysis were performed using SAS 12.0 statistical software. Significant differences among different treatments were determined by using Duncan’s multiple range test (DMRT) at the 95% confidence interval (p<0.001).

## Results

### Cloning of cecropin genes from *P*. *xylostella*

**Fig 1:**
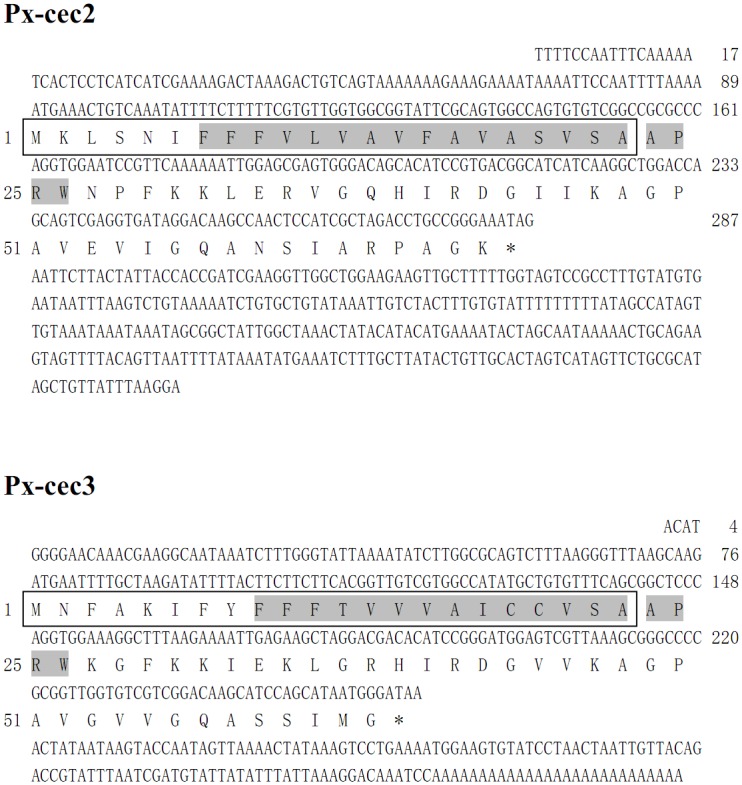
Nucleotide sequence of Px-cec2 and Px-cec3 cDNA from *P*. *xylostella* and their deduced amino acids. The putative signal peptide is boxed, while the mature peptide is indicated in bold type, while the predicted transmembrane domain is shaded. The stop codon is marked as an asterisk.

Two full-length cDNA sequences of Px-cec2 and Px-cec3 were isolated from transcriptome and 5′ and 3′ RACE from of fungi infected *P*. *xylostella* in Fig 1. The nucleotide sequence of Px-cec2 (GenBank accession No. GU391356) consisted of 198 bp open reading frame (ORF) encoding a predicted polypeptide of 65 amino acids, while the deduced mature protein (Px-cec2) comprised of 39 amino acid residues having a predicted molecular weight of 4.1 kDa and a theoretical isoelectric point (pI) of 10.44. Whereas, the nucleotide sequence of Px-cec3 (GenBank accession No. KF960048) contained 186 bp ORF with 35 amino acids and a predicted molecular mass of 3.59 kDa and 10.57 pI (Fig 1).

### Sequence analysis of *P*. *xylostella* cecropins

The multiple sequence alignment of cecropins from *P*. *xylostella* showed high conservation of the mature cecropins (S1 Fig). Identical amino acids of four peptides at the same position are shown with asterisk, indicating these positions to have a single, fully conserved residue, however, these four genes also showed dissimilar sites. The phylogenetic tree indicated homology of Px-cecs with several Lepidopteran’s cecropins such as cecropin D from *Agrius convolvuli* (Linnaeus) and *Spodoptera litura* (Fabricius), moreover, both Px-cec2 and Px013797 (*P*. *xylostella* Genome Database) showed maximum similarity with Px-cec1, suggesting the similar function (S2 Fig).

### qRT-PCR of *P*. *xylostella* cecropins

**Fig 2:**
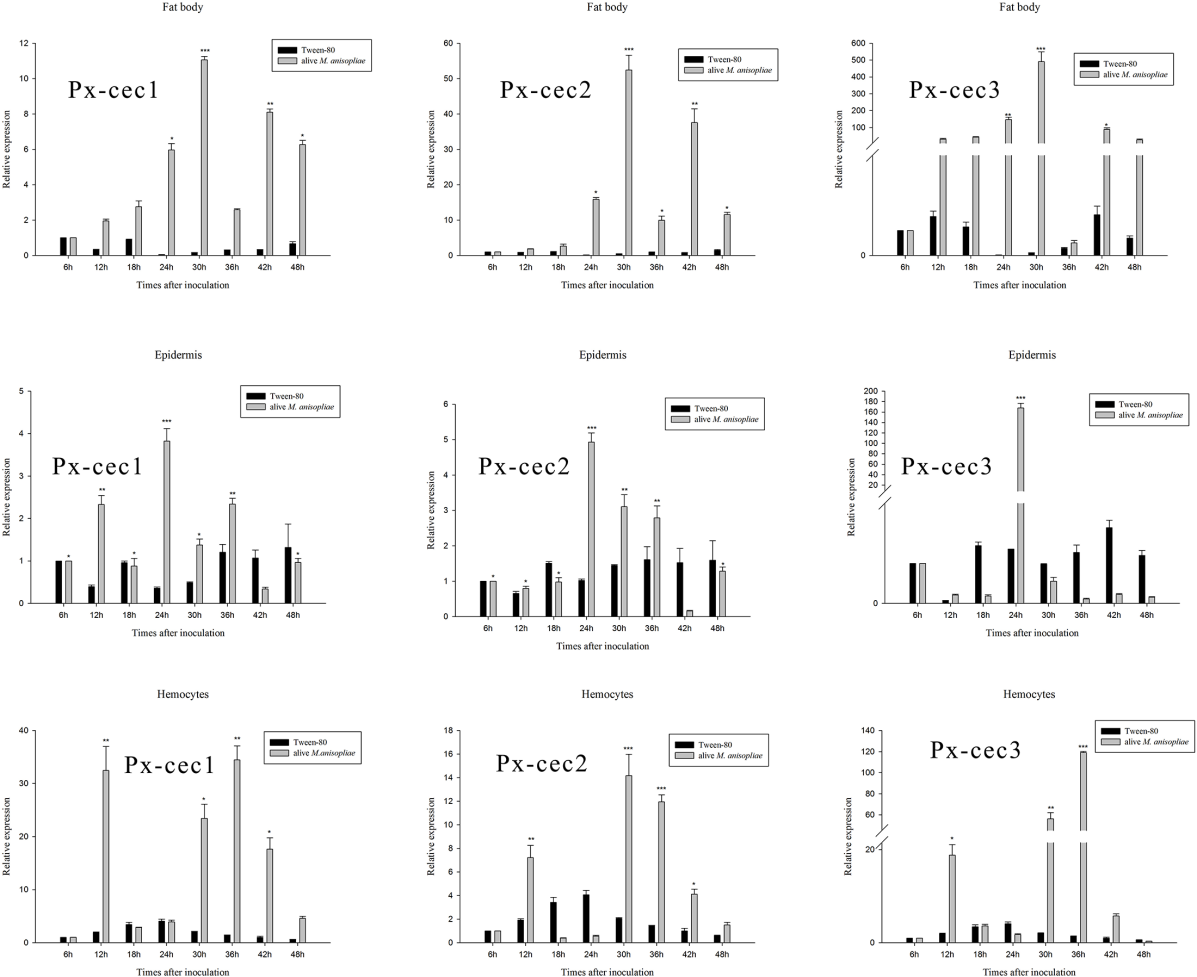
qRT-PCR analysis of relative expression of cecropin genes from fat body, epidermis, hemocytes after inoculation of *M*. *anisopliae*. Actin was used as an internal control. The mRNA levels of cecropins were highly expressed in 30 h after induction of *M*. *anisopliae* in fat body and 24 h in epidermis, Px-cec1, Px-cec2 and Px-cec3 showed maximum expression after 36 h, 30 h, 36 h in hemocytes, respectively. In addition, Px-cec3 depicted more sensitivity to *M*. *anisopliae* than Px-cec1 and Px-cec2. Relative expression levels of 6 h was arbitrarily set at 1. Three biological replications (n = 3) were conducted, and the 2^-ΔΔCt^ method was used to measure the relative transcription levels. Means with different number of asterisk are significantly different (P<0.05) (Duncan’s Multiple Range Test) among different time after treated with alive *M*. *anisopliae*. *: different with the lowest expression after treated with *M*. *anisopliae*; **: significant different with the lowest expression after treated with *M*. *anisopliae*; ***: highly significant different with the lowest expression after treated with *M*. *anisopliae*.

The qRT-PCR results indicated Px-cecs to be mainly expressed in fat body and epidermis after 30 and 24 h induction of *M*. *anisopliae*, respectively. Px-cec1 and Px-cec3 were highly expressed after 36 h, while Px-cec2 was highly expressed 30h after the inoculation of *M*. *anisopliae* in hemocytes (Fig 2). Moreover, the results from Fig 2 depicted the sensitivity of Px-cec3 to *M*. *anisopliae* with maximum expression in all tested tissues.

### RNAi effect on expressional level of Px-cecs

**Fig 3:**
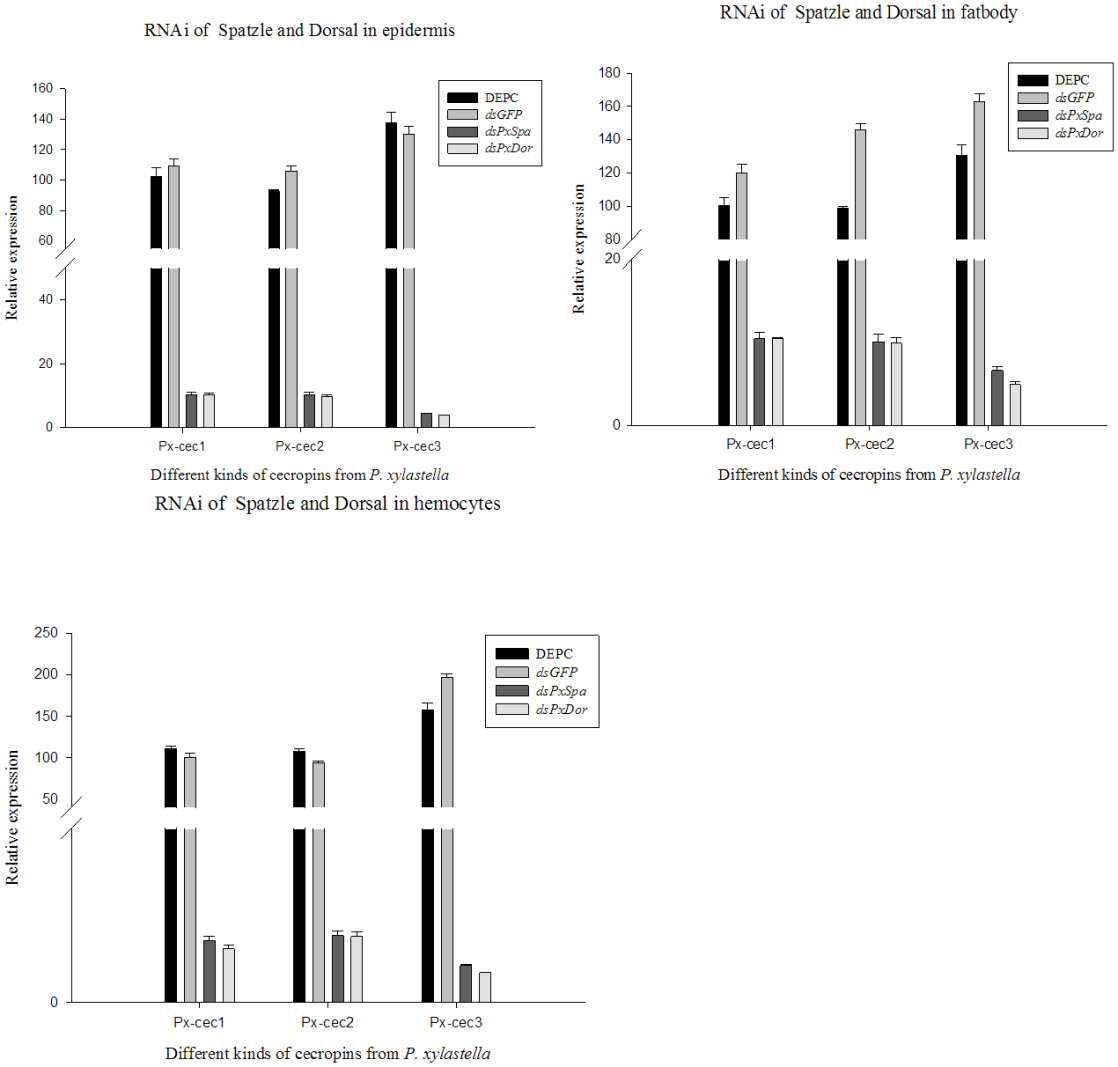
The knock-down of Spätzle and Dorsal independently by RNAi. qRT-PCR analysis of cecropins on fat body, epidermis and hemocytes in *P*. *xylostella* after 36 h of RNAi. The relative expression levels of cecropins mRNA was different after treatments. The mRNA level of Px-cec1 was remarkably decreased in fat body, Px-cec2 in hemocytes, while Px-cec3 was dropped rapidly as compared to other tissues. Actin was used as an internal control. Each bar represents the mean ± S.E. (n = 3).

**Fig 4:**
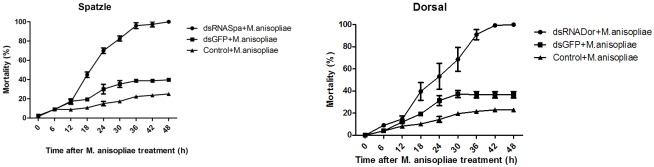
The analysis on susceptibility of *P*. *xylostella* larvae to alive *M*. *anisopliae* after knockdown of PxSpa and PxDor. After dsRNA treatment, the larvae were treated with *M*. *anisopliae* spores (1×10^9^ conidia/ml). Mortality was recorded every 6h. Each treatment was replicated three times and each treatment consisted of 30 larvae.

The qRT-PCR results revealed that the expression level of both Spätzle and Dorsal decreased significantly after 24 till 60 h (S3 and S4 Figs). On the other hand, Px-cecs were weakly detected in 36 h group. The mRNA level of Px-cec1 was remarkably decreased in fat body, Px-cec2 in hemocytes, while, Px-cec3 dropped rapidly in all three tissues (Fig 3). The results demonstrated cecropins to be presumably involved in the regulation by Toll pathways in *P*. *xylostella*. Meanwhile, after treated by dsRNA to Pxspa and Pxdor separately, which made the 4^th^ instar larvae more susceptible to *M*. *anisopliae* infection. Control larvae showed less than 60% mortality to *M*. *anisopliae* (1×10^9^ conidia/ml), while the larvae treated by dsRNA (Pxspa and Pxdor) showed significantly increased susceptibility and resulted in almost 100% mortality within 2 days post infection (Fig 4). The results suggested that Pxcecs are efficient factors regulated by Toll signal pathway against the fungal infection in *P*. *xylostella*.

### Expression and purification of cecropins

**Fig 5:**
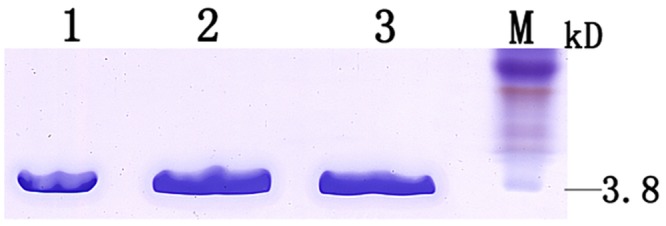
SDS-PAGE analysis of recombinant protein from Drosophila S2 cells. M: Kaleidoscope polypeptide weight protein marker; 1: recombinant protein of Px-cec1; 2: recombinant protein of Px-cec2; 3: recombinant protein of Px-cec3.The result showed that there was a single band corresponding to the expected size of 3.8 kDa.

**Fig 6:**
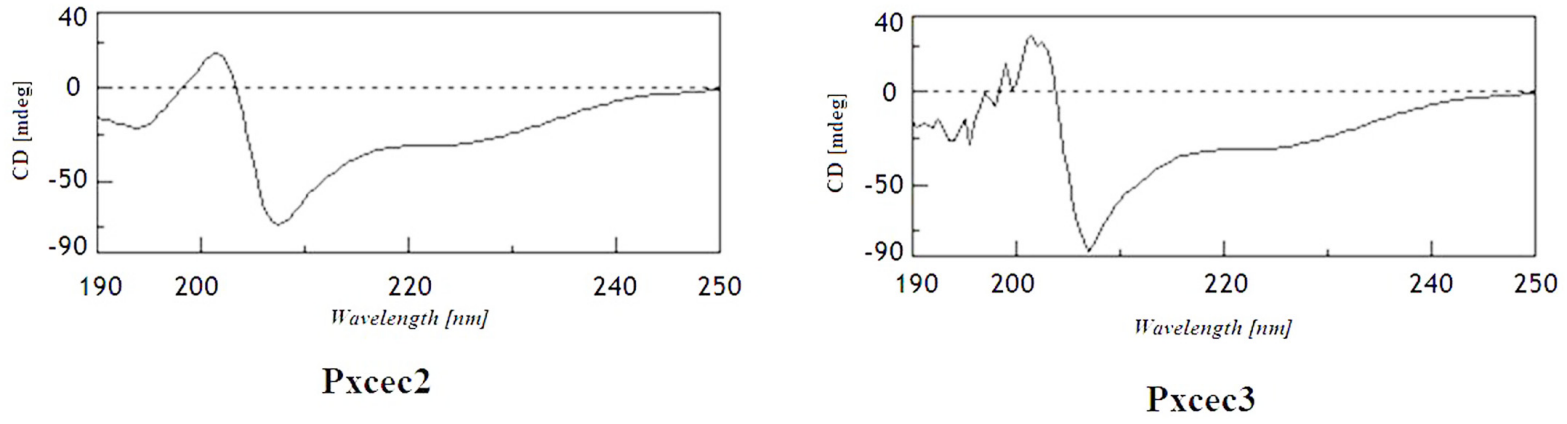
Analysis of Pxcec2 and Pxcec3 using far-UV CD spectra under 10 mM phosphate buffer (pH 7.2) at room temperature.

The cDNA fragment encoding mature Pxcecs were amplified by PCR and inserted into the *Drosophila* expression vector pMT/BiP/V5-HisA. Following transient transfection of *Drosophila* S2 cells with pMT-Pxcecs and induction expression with final concentration of 250μM copper sulfate, a recombinant protein was detected by SDS-PAGE in both medium and cell lysate. In order to purify the protein, stable transformant strains were selected by pcoBlast and purified recombinant cecropins were analyzed by Tricine-SDS-PAGE (Fig 5), which revealed that Pxcecs were purified to homogeneity with molecular mass of 3.8 kDa. The homogeneities were confirmed by MALDI-TOF-MS analysis (data not shown). The purified Pxcecs were also analyzed using far-UV CD at room temperature. The relative secondary structure contents of recombinant Pxcec2 and Pxcec3 mainly comprised of alpha-helices with some random coil in 10mM phosphate buffer (pH 7.2)(Fig 6), which were almost identical to the secondary structure of Px-cec1, reported previously by our lab [5].

### Antimicrobial activity assays of recombinant Pxcecs

Antimicrobial activity of recombinant Pxcecs were tested against several microbial strains, including Gram-negative, Gram-positive and fungi. The findings of minimal inhibitory concentration (MIC) showed that Pxcecs exhibited high antimicrobial activity against the tested strains (Table 2). Pxcecs illustrated a higher efficacy against Gram-negative than Gram-positive bacteria and fungi. Among the tested microorganisms, *E*. *coli* DH5α proved to be the most sensitive strain to Pxcecs, with MIC values of 0.1(Px-cec1), 0.5(Px-cec2) and 0.2(Px-cec3) μM, respectively. While for the fungi isolates, Pxcecs showed higher activity against *M*. *anisopliae*, with MIC values of 4.5(Px-cec1), 5.1(Px-cec2) and 6.5 (Px-cec3) μM, than another selected isolates of fungi.

**Table 2:** Antimicrobial activity of cecropins from *P*. *xylostella*.

Microorganisms	MIC (μM)		
	Px-cec1	Px-cec2	Px-cec3
**Gram-positive**			
*Staphylococcus aureus*	2.1	5.0	2.5
*Bacillus cereus*	2.6	3.5	2.7
**Gram-negative**			
*Escherichia coli* DH5α	0.1	0.5	0.2
*Pseudomonas fluorescent*	1.1	1.9	1.3
*Salmonella choleraesuis*	2.1	2.5	2.2
**Fungi**			
*Metarhizium anisopliae*	4.5	5.1	6.5
*Botrytis cinerea*	15.0	10.8	11.0
*Penicillium crustosum*	13.0	11.9	12.0
*Peronophythora litchi*	14.0	15.0	12.0
*Colletotrichum gloeosporioiees Penz*.	17.3	18.2	19.0
*Colletotrichum orbicucar*	12.5	25.0	20.0
*Fusarium oxysporum*	8.0	12.0	9.0

MIC, minimal growth inhibition concentrations; Px-cec1, the recombinant protein cecropin 1; Px-cec2, the recombinant protein cecropin 2 Px-cec3, the recombinant protein cecropin3

### Effect of cecropins on *M*. *anisopliae* spore morphology

**Fig 7:**
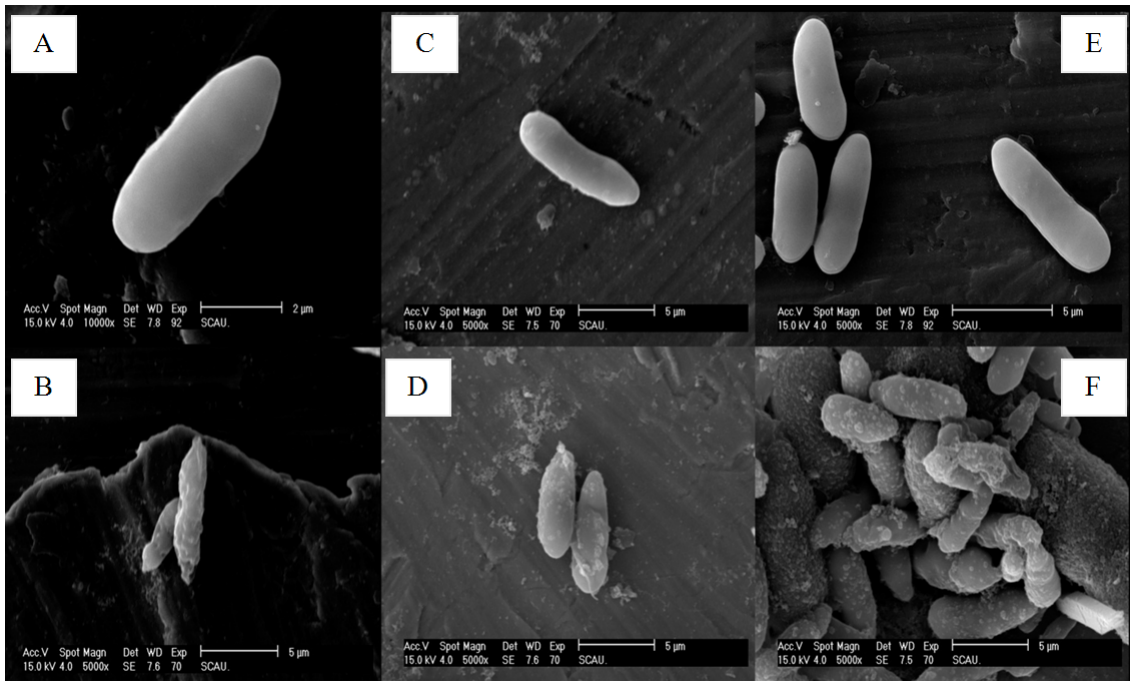
SEM analysis of the spore of *M*. *anisopliae* interacted with cecropins. A, C and E: CK (naive *M*. *anisopliae* spore); B, D and F: the spore of *M*. *anisopliae* interacted with Px-cec1, Px-cec2 and Px-cec3, respectively. The SEM analysis showed that *M*.*anisopliae* spore became short and wrinkled (B, D and F) after interacted with cecropins from *P*. *xylostella* as compared to the untreated spore (A, C and E), which had a bright and normal smooth surface.

**Fig 8:**
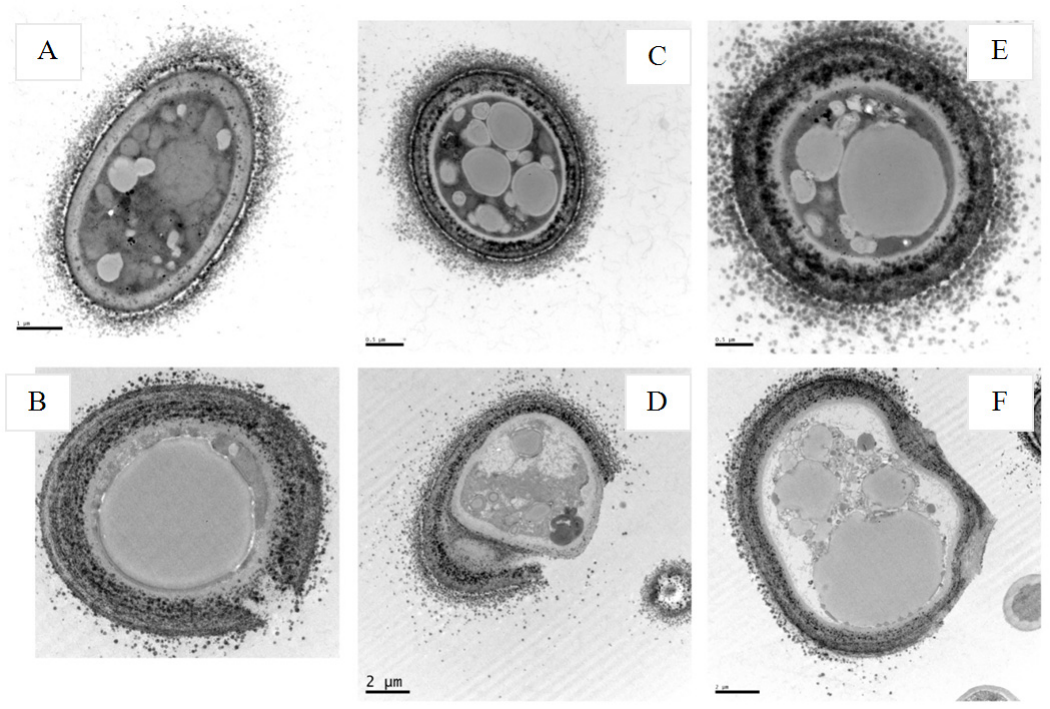
TEM showing the effect of cecropins from *P*. *xylostella* on the spores of *M*. *anisopliae*. A, C and E: CK (naive *M*. *anisopliae* spore); B, D and F: the spore of *M*. *anisopliae* interacted with Px-cec1, Px-cec2 and Px-cec3, respectively. The TEM images revealed that the cell membrane of the spore became wafery and the cellular cytoplasmic contents were dissolved and became vague (B, D and F). The entire cell membrane was disrupted, causing the cellular cytoplasmic contents to leak out after interaction with Px-cec1 and Px-cec2 (B and D), the untreated spore (A, C and E) had a bright and normal smooth surface and the cellular cytoplasmic content was clear.

The treatment of *M*. *anisopliae* spores with cecropins at 30 μM for 3 h, showed a few differences in the morphology. Cecropins induced spores remained short, while, the cell wall of the spores showed wrinkles and experienced blebbing as compared to the control (Fig 7). The morphological changes of *M*. *anisopliae* spore after treatment with 30μM Px-cecs were also examined using TEM, which showed that the cell membrane appeared to be wafery and the cellular cytoplasmic contents were dissolved and unclear. In addition, the entire cell membrane was disrupted, causing the cellular cytoplasmic contents to leak after interacted with Px-cec1, Px-cec2 and Px-cec3 (Fig 8). Conversely the untreated spores had a bright and normal smooth surface with clear cellular cytoplasmic contents.

## Discussion

Cecropins, the first antibacterial peptides discovered in animals containing α-helix[36,37]. In this study, two full length cDNA encoding Px-cec2 and Px-cec3 from *P*. *xylostella* were cloned and the recombinant proteins i.e., Px-cec1, Px-cec2 and Px-cec3 were purified from *Drosophila* S2 cells. The antibacterial activity assay showed recombinant proteins to have strong activity against *M*. *anisopliae*.

The cecropin genes of *D*. *melanogaster* and *Anopheles gambiae* have been categorized into four types i.e., A, B, C and D, while, *B*. *mori* cecropin genes have been divided into five types (A, B, C, D and E) [38]. However, according to the present study, all sequences from *P*. *xylostella* have been categorized into three types (Px-cec1, Px-cec2 and Px-cec3), moreover Px-cec2 and Px-cec3 are the first to be reported and characterized.

In *B*. *mori*, the cecropin A and B genes showed highest expression in the fat bodies and hemocytes, while, the cecropin D was expressed only in the fat bodies and hemocytes [39]. On the other hand, all functional genes were expressed, mainly in fat bodies after bacterial infection, although at different stages of development in *D*. *melanogaster* [23], but the expression of cecropins after fungal induction has not been reported in *P*. *xylostella*. To understand the role of cecropins in mRNA level against fungal induction in *P*. *xylostella*, three tissues i.e., fat body, epidermis and hemocytes were isolated from *M*. *anisopliae* infected larvae. qRT-PCR investigation showed that Px-cec1 and Px-cec3 were mainly expressed in fat body, while Px-cec2 in hemocytes (Fig 2). Interestingly, the transcript level of Px-cec3 was higher than those of Px-cec1 and Px-cec2 in the three selected tissues. Based on above results, *P*. *xylostella* cecropins displayed specific expressional profiles at different time in the three selected tissues, which demonstrated Px-cec3 to take a predominant position to resist the fungal infection. Moreover, the time required to achieve the highest expression in epidermis was earlier than fat body and hemocytes revealing *M*. *anisopliae* adherence to epidermis at first, then to fat body and hemocytes.

Insects and microbes partially share the same environment, including *D*. *melanogaster* and *B*. *mori*. *D*. *melanogaster* has evolved sensitive mechanisms for pathogen recognition and various strategies to defend against bacteria, fungi, parasites and viruses [40]. Expression of AMP genes in *D*. *melanogaster* is regulated by the two signal transduction pathways [41]. Imd pathways may be taking the roles to regulate the expression of diptericins, while, Toll pathways transduct the signals to trigger the expression of cecropins, drosomycins etc. At present, the Toll signaling pathways has been well studied in *D*. *melanogaster*, but less characterized in other insect species [41], meanwhile, Spätzle and Dorsal have been reported to activate Toll pathways in *D*. *melanogaster* [42,43]. To verify insect pests Toll pathways in vivo, RNAi in *P*. *xylostella* was performed. Our results confirmed that cecropins were mainly regulated by Toll pathways and participate in innate immunity after *M*. *anisopliae* infection in *P*. *xylostella*. Importantly, qRT-PCR indicated that Px-cec3 owned more sensitivity towards *M*. *anisopliae* with maximum expression in fat body, epidermis and hemocytes. In addition, the mRNA level of Px-cec1 was strongly decreased in hemocytes, while Px-cec2 and Px-cec3 were notably decreased in the fat body which depicted cecropins to exhibit tissue-specific expression in *P*. *xylostella*.

Purified Px-cec1 has already been obtained by constructed vector pET-32a (+) (Novagen, USA) in the previous study [5]. In the present study, the recombinant proteins Px-cec1, Px-cec2 and Px-cec3 were expressed and purified from *Drosophila* S2 cells, which enhanced the quality of protein, while the activity of Px-cec1 against pathogenic bacteria has already been tested in our lab. In this research, seven fungi were added for testing. Recombinant protein Pxcecs showed strong antibacterial activity against Gram-positive, Gram-negative bacteria and fungi, which affirmed cecropins to have broad-spectrum antibacterial property. Recombinant cecropins showed lower MIC values against Gram-negative bacteria than other tested strains (Table 2). In comparison to the cecropins tested in our lab, Pxcecs illustrated higher antibacterial activity against *P*. *crustosum*, in contrast to already reported cecropins against *P*. *crustosum* from *Musca domestica* [44].

In the previous study, the detail mechanism of microbial cell lysis by the antimicrobial peptides is not well understood. It is believed that antimicrobial peptides may form an ion channel or pore on the cell membrane prior to cell death[45,46]. Cecropins interacted with bacterial membranes resulting in the formation of ion channels, so as to kill the microorganisms[47]. SEM and TEM of Px-cec1 on *S*. *aureus* have been already reported from our laboratory [5].In the current study, to elucidate the mechanisms of Px-cecs against fungi, we used SEM and TEM to assay the morphological changes of the spores of *M*. *anisopliae* induced by cecropins treatment. We found that cell surface of spores treated with cecropins was damaged and disrupted and gross leakage of cytoplasmic contents was also observed. Our results suggested that the fungi spores membrane is an important target of antimicrobial peptide and also illustrated cecropins playing crucial role in innate immunity of *P*. *xylostella*.

In short, this study is the first to our knowledge to examine the biological activity of Px-cec2 and Px-cec3 and widen the horizon of Px-cec1. The results of qRT-PCR suggested that Px-cec1, Px-cec2 and Px-cec3 were differentially expressed after induction of *M*. *anisopliae*. Recombinant proteins Pxcecs displayed high resistance towards all tested microorganisms. Based on our results, cecropins resisted the infection of fungi and for the effective fungal applications as pesticides or the blockage of signal transduction pathways in *P*. *xylostella*, it is necessary to reduce its ability to resist against entomopathogenic fungi, which will in turn improve the effectiveness of fungi.

## Supporting Information

S1 Fig Alignment of the amino acid sequences of Px-cec1, Px-cec2, Px-cec3 and Px013797 (derived from *P*. *xylostella* Genome Database). Conserved amino acid residues among four genes (black background), identical residues among three genes (pink background), and same residues among two genes are shown on blue background.
(TIF)

S2 Fig A phylogenetic tree of the cecropin amino acid sequences by neighbor joining method. *Drosophila melanogaster* cecropin C (AAB82507); *Drosophila melanogaster* cecropin B (AAF57027.1); *Aedes aegypti* cecropin A (AAF59831); *Manduca sexta* cecropin 6 (AAO74638); *Agrius convolvuli* cecropin D precursor (ACX37671); *Hyalophora cecropia* cecropin partial(AAP93872); *P*. *xylostella* cecropin 1 (ADA13281); *P*. *xylostella* cecropin 2 (ADC54851); Px013797(derived from *P*. *xylostella* Genome Database); *Pseudoplusia includens* cecropin A (AAR99379); *Papilio xuthus* cecropin (ACR82292); *Bombyx mori* cecropin precursor(NP-001037392); *P*. *xylostella* cecropin 3 (KF960048); *Hyphantria cunea* cecropin A3 (AAB39003); *Hyphantria cunea* cecropin A (AID51414); *Bombyx mori* cecropinB precursor (NP-001096031); *Spodoptera litura* cecropin D partial(ABQ51092); *Hyalophora cecropia* cecropin B(AAA29184); *Trichoplusia ni* cecropin B (ABV68872); *Helicoverpa armigera* cecropin (AAX51304); *Antheraea pernyi* cecropin B(P01509); *Tribolium castaneum* cecropin 2 precursor (NP-001164146).
(TIF)

S3 Fig The RNAi result of Spätzle. qRT-PCR analysis of Spätzle after RNAi on fat body, epidermis and hemocytes in *P*. *xylostella* from 12 h to 60 h. The relative expression levels of Spätzle mRNA was different after treatments, Means with two asterisks are statistically different (*p<0*.*001*) (Duncan’s Multiple Range Test) among treatments; Actin was used as an internal control. Each bar represents the mean ± S.E. (n = 3).
(TIFF)

S4 Fig The RNAi result of Dorsal. qRT-PCR analysis of Dorsal after RNAi on fat body, epidermis and hemocytes in *P*. *xylostella* from 12 to 60 h. The relative expression levels of Dorsal mRNA was different after treatments, Means with two asterisks are statistically different (*p<0*.*001*) (Duncan’s Multiple Range Test) among treatments; Actin was used as an internal control. Each bar represents the mean ± S.E. (n = 3).
(TIF)
