# Supplementary figures and images for: Cecropins from Plutella xylostella and Their Interaction with Metarhizium anisopliae
Source: PLoS One. 2015 Nov 6;10(11):e0142451. doi: 10.1371/journal.pone.0142451 (PMC4636316; doi:10.1371/journal.pone.0142451)

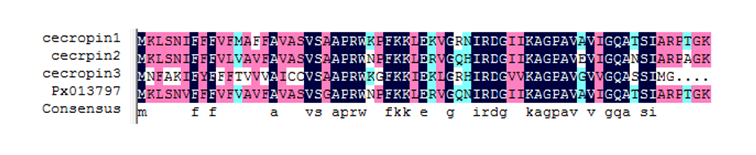

Supplement: S1 Fig — Conserved amino acid residues among four genes (black background), identical residues among three genes (pink background), and same residues among two genes are shown on blue background. (TIF) [file pone.0142451.s001.tif]

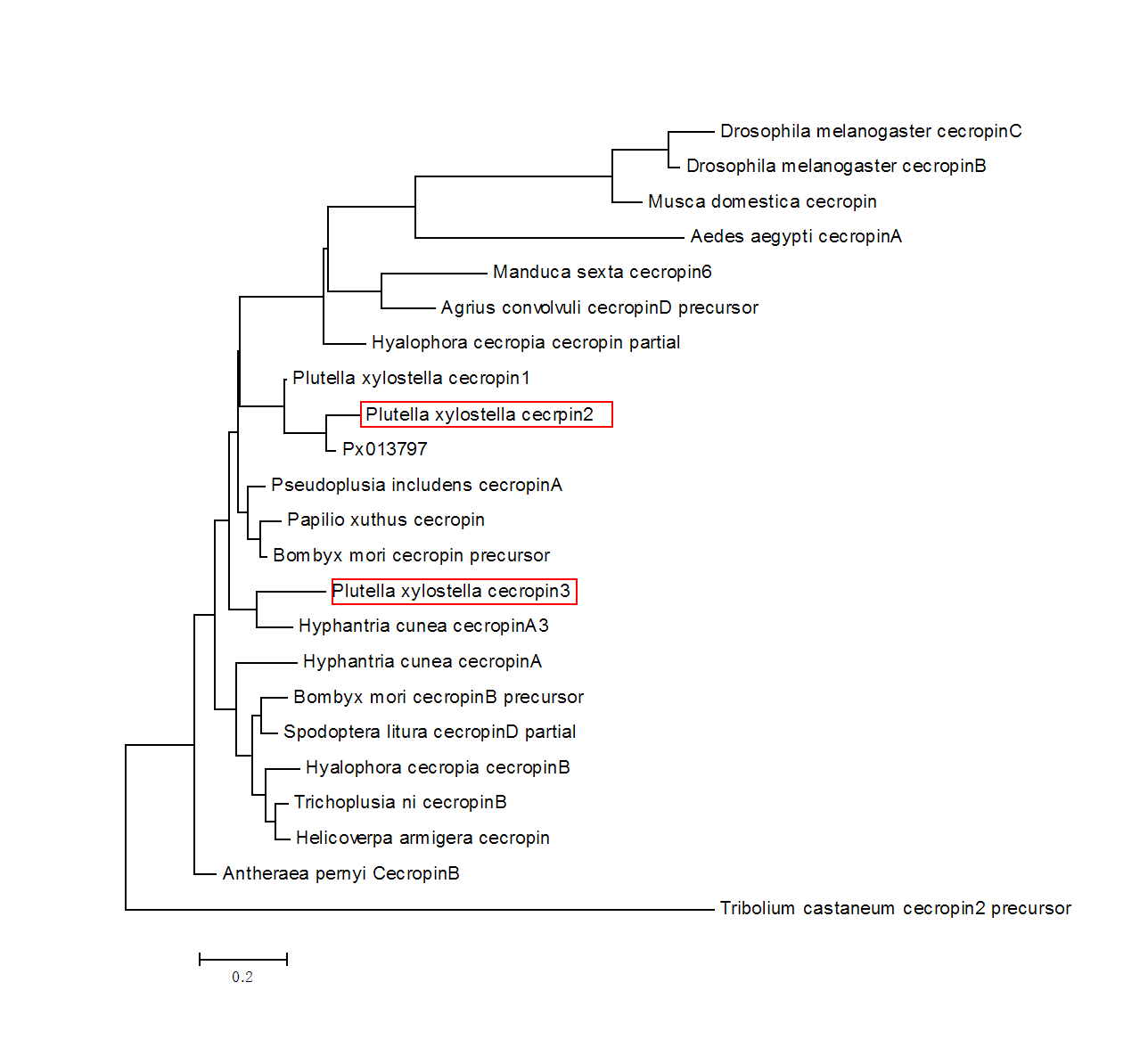

Supplement: S2 Fig — Drosophila melanogaster cecropin C (AAB82507); Drosophila melanogaster cecropin B (AAF57027.1); Aedes aegypti cecropin A (AAF59831); Manduca sexta cecropin 6 (AAO74638); Agrius convolvuli cecropin D precursor (ACX37671); Hyalophora cecropia cecropin partial(AAP93872); P. xylostella cecropin 1 (ADA13281); P. xylostella cecropin 2 (ADC54851); Px013797(derived from P. xylostella Genome Database); Pseudoplusia includens cecropin A (AAR99379); Papilio xuthus cecropin (ACR82292); Bombyx mori cecropin precursor(NP-001037392); P. xylostella cecropin 3 (KF960048); Hyphantria cunea cecropin A3 (AAB39003); Hyphantria cunea cecropin A (AID51414); Bombyx mori cecropinB precursor (NP-001096031); Spodoptera litura cecropin D partial(ABQ51092); Hyalophora cecropia cecropin B(AAA29184); Trichoplusia ni cecropin B (ABV68872); Helicoverpa armigera cecropin (AAX51304); Antheraea pernyi cecropin B(P01509); Tribolium castaneum cecropin 2 precursor (NP-001164146). (TIF) [file pone.0142451.s002.tif]

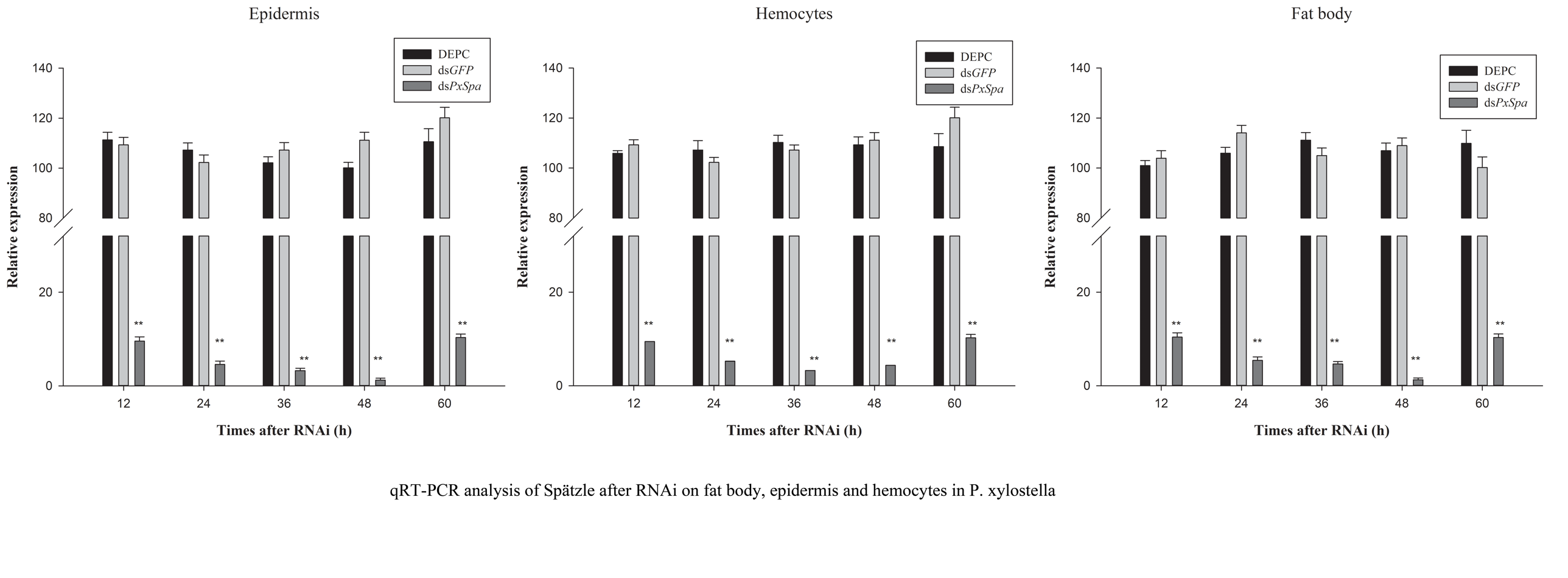

Supplement: S3 Fig — qRT-PCR analysis of Spätzle after RNAi on fat body, epidermis and hemocytes in P. xylostella from 12 h to 60 h. The relative expression levels of Spätzle mRNA was different after treatments, Means with two asterisks are statistically different (p<0.001) (Duncan’s Multiple Range Test) among treatments; Actin was used as an internal control. Each bar represents the mean ± S.E. (n = 3). (TIFF) [file pone.0142451.s003.tiff]

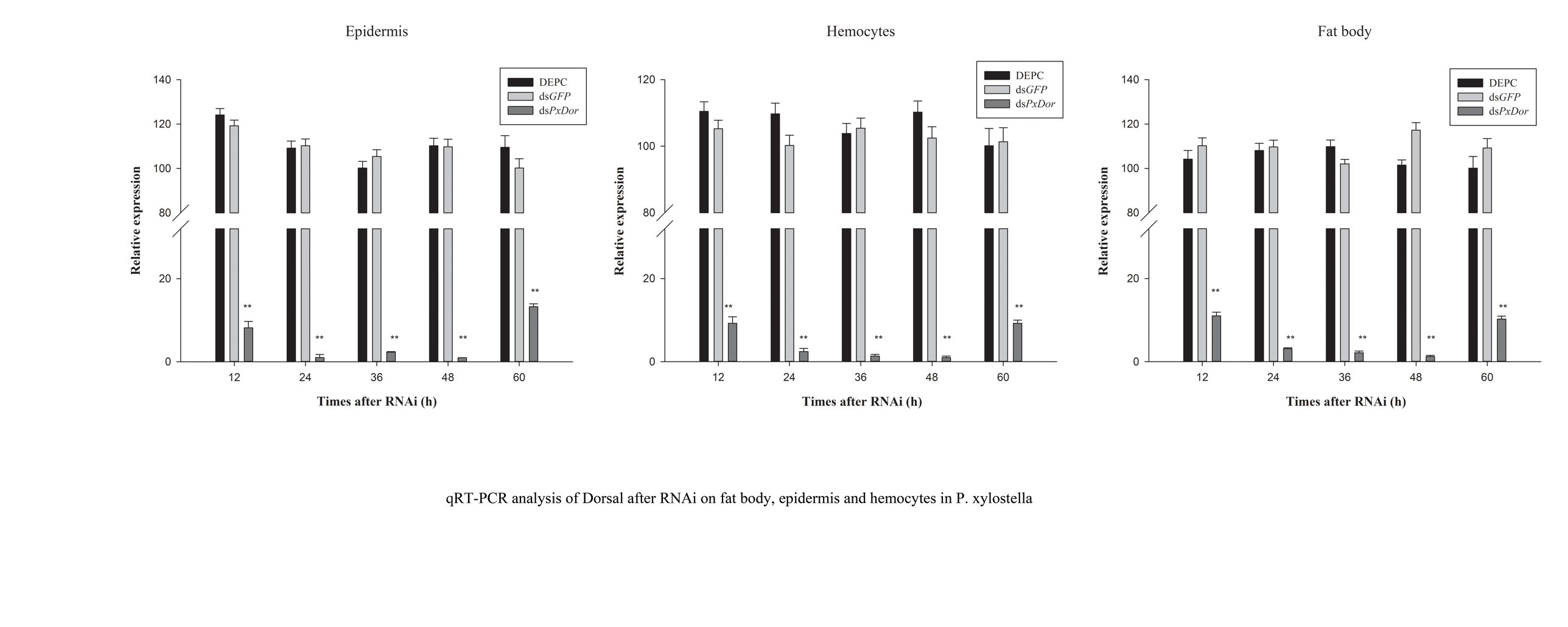

Supplement: S4 Fig — qRT-PCR analysis of Dorsal after RNAi on fat body, epidermis and hemocytes in P. xylostella from 12 to 60 h. The relative expression levels of Dorsal mRNA was different after treatments, Means with two asterisks are statistically different (p<0.001) (Duncan’s Multiple Range Test) among treatments; Actin was used as an internal control. Each bar represents the mean ± S.E. (n = 3). (TIF) [file pone.0142451.s004.tif]
